# Malignancy rates in thyroid nodules: a long-term cohort study of 17,592 patients

**DOI:** 10.1530/ETJ-22-0027

**Published:** 2022-05-30

**Authors:** M Grussendorf, I Ruschenburg, G Brabant

**Affiliations:** 1 Department of Internal Medicine, University Hospital, Düsseldorf, Germany; 2 MVZ Wagnerstibbe Center for Cytology and Pathology, Einbeck, Germany; 3 Department of Diabetes, Endocrinology and Gastroenterology, School of Medical Sciences, University of Manchester, Manchester, UK

**Keywords:** goiter, thyroid nodules, FNAC, thyroid malignancy, follow-up

## Abstract

**Objectives:**

Ultrasound diagnosis of thyroid nodules has greatly increased their detection rate. Their risk for malignancy is estimated between 7 and 15% in data from specialized centers which are used for guidelines recommendations. This high rate causes considerable anxiety to patients upon first diagnosis. Here, we retrospectively analyzed the malignancy rate of sonographically diagnosed nodules larger than 1 cm from a primary/secondary care center when long-term longitudinal follow-up was included.

**Patients/methods:**

In the study, 17,592 patients were diagnosed with a thyroid nodule larger than 1 cm, of whom 7776 were assessed by fine-needle aspiration cytology (FNAC) and 9816 by sonography alone. 9568 patients were initially discharged due to innocent results of FNAC and/or ultrasound. In 1904 patients, definitive histology was obtained, and 6731 cases were included in the long-term follow-up (up to 23 years, median 5 years).

**Results:**

Malignancy was histologically confirmed in 189 patients (1.1% of all) when excluding accidentally diagnosed papillary microcarcinomas. 155 were diagnosed during the first year of management, 25 in years 2–5 of follow-up, 9 in years 6–10 and nil in 1165 patients followed beyond 10 years.

**Conclusions:**

The malignancy rate of thyroid nodules from primary/secondary care was much lower than that previously reported. During follow-up for more than 5 years, their rate rapidly dropped to less than 1/1000 cases. This low malignancy rate may help to reassure patients first confronted with the diagnosis of a thyroid nodule, substantially reduce their anxiety and avoid unwarranted diagnostic and therapeutic procedures.

## Introduction

Inclusion of thyroid ultrasound in the routine diagnostic workup greatly increased the number of thyroid nodules detected (1, 2, 3, 4, 5). According to epidemiological studies, a prevalence of up to 65% of the adult population can be expected (3, 5, 6). If we base the rate of malignancy (ROM) of these nodules on data from current guidelines, between 7 and 15% of the nodules bear a malignancy (7, 8). As these data mainly reflect nodules suspected on the basis of ultrasound (US) criteria and possibly fine-needle aspiration cytology (FNAC), malignancy rates may be overestimated and will not necessarily reflect the chances of a nodule being first diagnosed by palpation or imaging methods. On the other hand, ROM may as well be underestimated because some thyroid cancers will be initially missed and will only be detected during long-term follow-up due to their low growth rates.

The diagnosis of a new thyroid nodule will cause anxiety in the patient (9, 10) and may well lead to further diagnostic procedures or even surgical clarification. Thus, a clear estimate of the true ROM in a primary diagnostic setting could minimize overreaction, either on the part of the patient or the diagnosing physician, and thus avoid potentially harmful intervention being undertaken.

To decrease preselection, we evaluated the ROM of all patients seen consecutively in our primary/secondary endocrine care center with a first diagnosis of a thyroid nodule larger than 1 cm in diameter (TN >1 cm), either following initial diagnosis by us or upon referral by a general practitioner. In addition, we longitudinally followed non-operated patients for up to 23 years (median: 5 years) to best capture ROM in potentially slow-growing and difficult to diagnose nodules.

## Methods

### Patients

A total of 17,592 patients with TN >1 cm sequentially diagnosed by US between March 1989 and April 2013 at the outpatient Endocrine Center Stuttgart, Germany, were evaluated retrospectively (see Table 1). The center was headed by a thyroid specialist (MG) and included a team of three fully qualified endocrinologists and two internal medicine physicians throughout the study period.

**Table 1:** Age and gender distribution of all patients with thyroid nodules larger than 1 cm.

Age (years)	Female (*n*)	Male (*n*)	All (*n*)	% of cohort
≤30	1001	132	**1133**	6.4
**31–50**	5645	1206	**6851**	38.9
**51–70**	5935	1674	**7609**	43.3
>70	1611	388	**1999**	11.4
**All**	14,192	3400	**17,592**	100.0

All data were pseudonymized prior to evaluation. The study was approved by the Ethics Committee of the Bavarian State Chamber of Physicians (query 2018/195).

### Ultrasound examinations

Five ‘generations’ of US equipment were used between 1989 and 2013: Picker CS9000 (transducer: 3.5 and 5.0 MHz), Picker CS9150 MACS (transducer: 3.5 and 7.5 MHz), Toshiba Nemio 20 (transducer: 3.5 and 7.5 MHz), Toshiba Nemio XG MK1 (transducer: 3.5 and 7.5 MHz (Duplex available)) and Hitachi HI Vision Avius (transducer: 3.5 and 10.0 MHz (Duplex and Elastography available)).

Thyroid sonography was used throughout the study for a highly standardized assessment and recording of thyroid structure as well as the volume, structure and metrics of thyroid nodules. Suspicion of malignancy was based on the presence of at least one of the following criteria: hypoechoic pattern, irregular margins and/or microcalcifications. We further selected nearly all scintigraphically hypofunctioning nodules for FNAC.

### FNAC

Patients with a nodule larger 1 cm and one or more sonomorphological criteria suspicious for malignancy (see above) were offered FNAC (for details see Supplementary methods, see section on supplementary materials given at the end of this article).

### Management and follow-up

A total of 1293 patients with TN >1 cm were sent to surgery after initial diagnosis and 9568 were discharged because of unsuspicious US and/or FNAC findings. A group of 6731 patients (38.3% of all) with low sonomorphological suspicion for malignancy including patients classified as Bethesda Class 2 (BC2) in FNAC (11) were longitudinally followed. As some patients needed repeat FNAC and had to wait for surgery, we defined the first year of diagnosis and treatment as ‘initial year of management’ (IYM). Duration of follow-up was calculated as the difference between the first and last visit of a patient. The timing of follow-up visits was governed by clinical needs. Thyroid surgery and histopathological assessment were performed in local referral centers.

**Figure 1:**
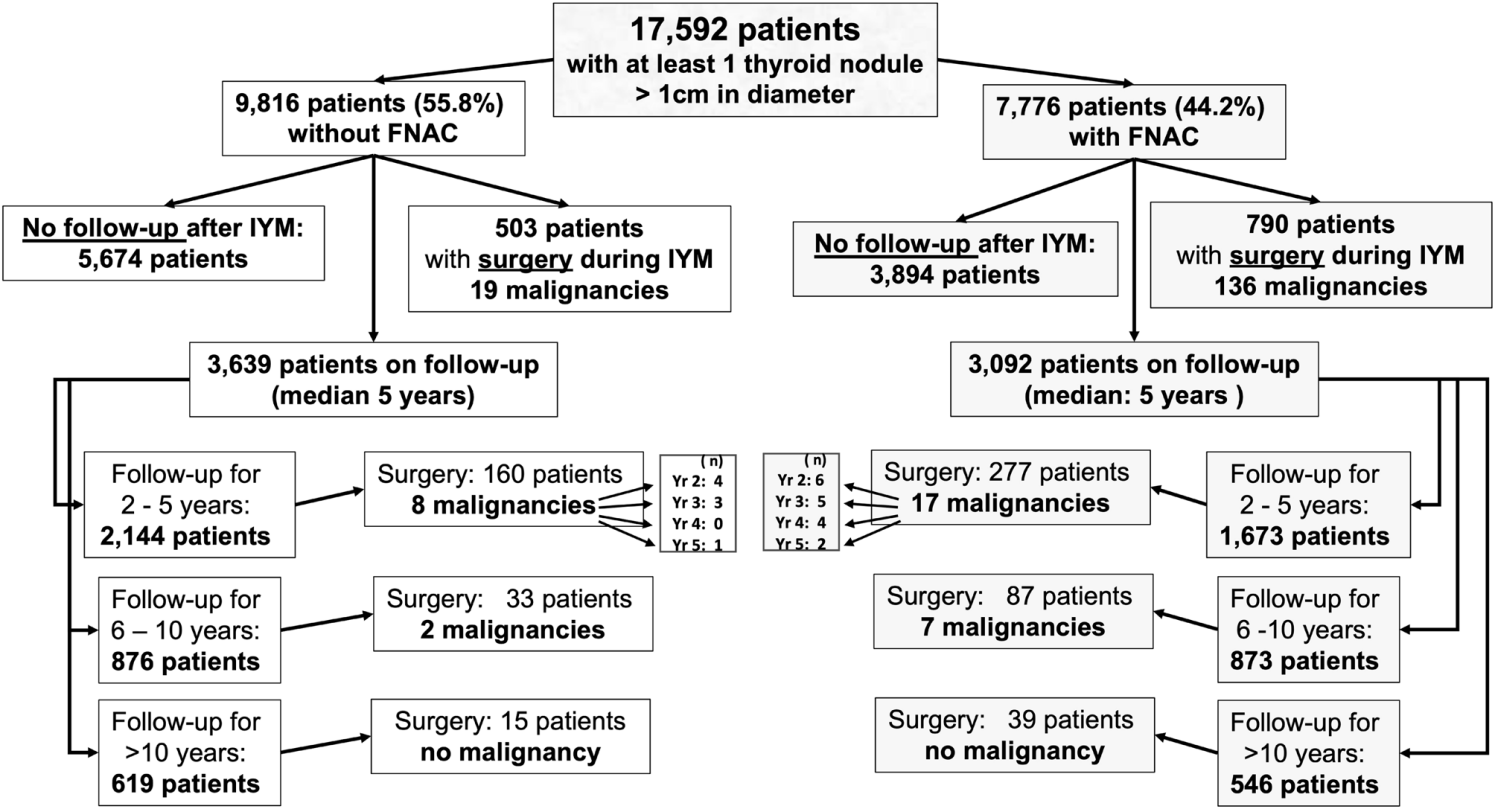
Study flow sheet.

A detailed flowchart showing the distribution and timing of long-term follow-up in patients with or without FNAC is provided in Fig. 1.

### Statistical analysis

All histologically proven malignancies but not patients histologically diagnosed with a papillary microcarcinoma (mPTC) were included in the calculation of the ROM which was defined as the percentage of patients receiving a diagnosis of a thyroid malignancy divided by all patients with TN >1 cm.

Positive predictive value (PPV) and negative predictive value (NPV) of initial FNAC results were calculated for each Bethesda class (BC). PPV was calculated for all malignancies, using the definition: true-positive/true-positives plus false-positive results, and NPV was defined by true-negative/true-negative plus false-negative results (for details see Supplementary methods).

## Results

All 17,592 TN >1 cm patients underwent clinical investigation, US and thyroid function tests. In 9816 patients, sonomorphological suspicion of malignancy was too low to justify FNAC, whereas 7776 patients were subjected to single or repeated FNAC (44.2% of all TN >1 cm patients (see Fig. 1)).

### Total ROM and TNM classification of all malignancies

Malignancy was histologically verified in 189 of all 17,592 TN >1 cm patients, corresponding to a ROM of 1.1% (Table 2). When stratified by age, only the risk of young patients under 30 years of age appears to be increased (ROM: 2.8%; see Table 3). Table 4 relates ROM to the presenting thyroid disorder.

**Table 2:** Histological findings in patients with thyroid malignancies in relation to gender and TNM classification.

Tumor type	Female (*n*) (% of all)	Male (*n*) (% of all)	All (*n*) (%)	TNM classification	T-class	All (*n*) (% of all malignancies)	N1 (*n*) (%)	M1 (*n*) (%)
PTC	78 (58.6)	31 (55.4)	109 (57.7)					
FTC	22 (16.5)	13 (23.2)	35 (18.5)	All malignancies	1	40 (21.2)	8	2
FVPTC	6 (4.5)	0	6 (3.2)		2	78 (41.3)	12	0
ATC	3 (2.3)	1 (1.8)	4 (2.1)		3	29 (15.3)	14	6
MTC	14 (10.5)	7 (12.5)	21 (11.1)		4	25 (13.2)	12	4
Malignant lymphoma	4 (3.0)	0	4 (2.1)		No data	17 (9.0)		
Metastases of other Ca’s	6 (4.5)	4 (7.1)	10 (5.3)					
All	133 (70.4)	56 (29.6)	189 (100)	All patients		189 (100)	46 (24.3)	12 (6.3)

ATC, anaplastic thyroid carcinoma; Ca, carcinoma; FTC, follicular thyroid carcinoma; FVPTC, follicular variant of papillary thyroid carcinoma; MTC, medullary thyroid carcinoma; PTC, papillary thyroid carcinoma; TNM, tumor lymph node metastasis.

**Table 3:** Age distribution of all patients and of ROM within the age groups.

Age (years)	All patients with TN >1 cm			
	Pts. (*n*)	% of all	Pts. with malign. (*n*)	ROM (%)
≤30	1133	6.4	32	2.8
31–50	6851	38.9	74	1.1
51–70	7609	43.3	66	0.9
>70	1999	11.4	17	0.9
All	17,592	100.0	189	1.1

malign., malignancy; pts., patients; ROM, rate of malignancy; TN >1 cm, thyroid nodule larger than 1 cm in diameter.

**Table 4:** Underlying diagnoses of patients and distribution of ROM within these cohorts

Diagnosis	Female (*n*)	Male (*n*)	All (*n*)	% of all	Pts. with malignancies	ROM (%)
Euthyroid goiter	11,109	2851	13,960	79.4	171	1.3
Graves’ disease	356	56	412	2.3	2	0.5
Autoimmune thyroiditis	906	68	974	5.5	5	0.5
Toxic adenoma	1540	373	1913	10.9	1	0.05
Disseminated autonomy	275	48	323	1.8	0	0
Other (metastases)	6	4	10	0.1	10	100.0
**All**	**14,192**	**3400**	**17,592**	**100.0**	**189**	**1.1**

pts., patients; ROM, rate of malignancy.

**Table 5:** Published rates of malignancy (calculated either as percent per patients or percent per nodules) and our findings.

Calculation per patients with thyroid nodules	All patients with nodular goiter (*n*)	Patients with FNAC (*n*)	Operated patients (*n*)	Patients with malignancies (*n*)	ROM of all pts. with FNAC (%)	ROM of all operated patients (%)	ROM of all pts. with nodules (%)
**Calculation per thyroid nodules**	**All nodules**(*n*)	**Nodules with FNAC** (*n*)	**Operated nodules** (*n*)	**Nodules with malignancies** (*n*)	**ROM of all nod. with FNAC** (%)	**ROM of all operated nodules** (%)	**ROM of all nodules** (%)
This study	17,592	7776	1904	189	2.4	9.9	1.1
Frates *et al.* (2006) (23)	Approx. 3200	1985		295	14.9		9.2
Angell *et al.* (2019) (20)		9967		1625	16.3		
Ng *et al.* (2021) (21)		2207		279	12.6		
Krauss *et al.* (2016) (13)		5574	634	220	3.9	34.7	
Liu *et al.* (2017) (15)		2838	791	673	23.7	85.1	
Reuters *et al.* (2018) (17)		980	418	140	14.3	33.5	
Thewjitcharon *et al.* (2019) (19)		2735	188	80	2.9	42.6	
Angell *et al.* (2019) (20)	20,001	14,389	4347	1760	12.2	40.5	8.8
Metaanalytical data:							
Bongiovanni *et al.* (2012) (12)		25,445	6362	2150	8.4	33.7	
Singh Ospina *et al.* (2016) (22)		80,079	15,641	4166	5.2	26.6	

FNAC, fine-needle aspiration cytology; pts., patients; ROM, rate of malignancy.

The histological diagnosis of malignant nodules was divided into 109 papillary thyroid carcinomas (PTC), 35 follicular thyroid carcinomas (FTC), 6 follicular variants of papillary thyroid carcinoma (FVPTC), 4 anaplastic thyroid carcinomas (ATC), 21 medullary thyroid carcinomas (MTC), 4 malignant lymphomas and 10 metastases of an extrathyroidal malignancy. 46 patients (24%) had lymph node manifestations and 12 patients (6%) had distant metastases.

In addition, the histological workup accidentally revealed a further 38 patients with mPTC unrelated to the sonographically suspicious nodule.

### Malignancies in patients subjected to FNAC

Details of the FNAC results are discussed in the supplement in relation to the corresponding histological diagnosis. They favorably compare to previously published data (11, 12, 13, 14, 15, 16, 17, 18, 19, 20, 21) (see Supplementary Tables 1, 2, 3 and 4).

633 BC1 samples showed non-informative results (8.1% of all FNACs); additionally in 1004 BC1 patients with pure cysts fluid was aspirated for subsequent cytology. 14 malignancies in nondiagnostic cases were classified as BC1 and 3 malignancies were histologically confirmed in patients with pure cysts (for details see Supplementary results and discussion).

The vast majority (5839 patients: 75.1% of all patients with FNAC) were classified as BC2. Of these, 763 subsequently received surgery, mainly for multinodular goiter or a suspected malignancy despite a BC2 result in FNAC with the histological diagnosis of malignancy in 29 cases (for details see Supplementary results and discussion).

114 patients initially classified as BC3-BC6 were diagnosed with a malignancy including 34 of 211 cases with BC3 and BC4, 18 of 27 cases with BC5 and all 62 patients with BC6 (see Supplementary Table 1).

A total of 3092 patients received long-term follow-up after FNAC due to persistent but low sonomorphological suspicion of malignancy (see Fig. 1).

### Malignancies in patients not subjected to FNAC

Among the 9816 TN >1 cm patients not subjected to FNAC, 9 patients refusing FNAC were directly operated owing to a high suspicion for malignancy which was subsequently confirmed (5 PTC, 3 FTC and 1 FVPTC). Serum calcitonin levels were elevated in 10 patients, indicating MTC which was verified histologically in all of them. A total of 484 patients were assigned for surgery during IYM owing to multinodular goiter with none of them histologically malignant. Of the 3639 patients included in the long-term follow-up, 208 were operated during the follow-up (mostly because of large or growing goitre), 10 of whom were found to have a malignancy in a nodule >1 cm (6 PTC, 1 FTC, 2 MTC and 1 lymphoma). The remaining cases consisted of patients discharged because they lacked any sonomorphological sign of malignancy or were lost to follow-up (see Fig. 1).

### Malignancies detected during initial management and follow-up

Malignancies were histologically confirmed during IYM in 155 patients with and without FNAC (91 PTCs, 25 FTCs, 18 MTCs. 4 FVPTCs, 4 ATCs, 10 metastases and 3 lymphomas; ROM: 0.88%).

**Figure 2:**
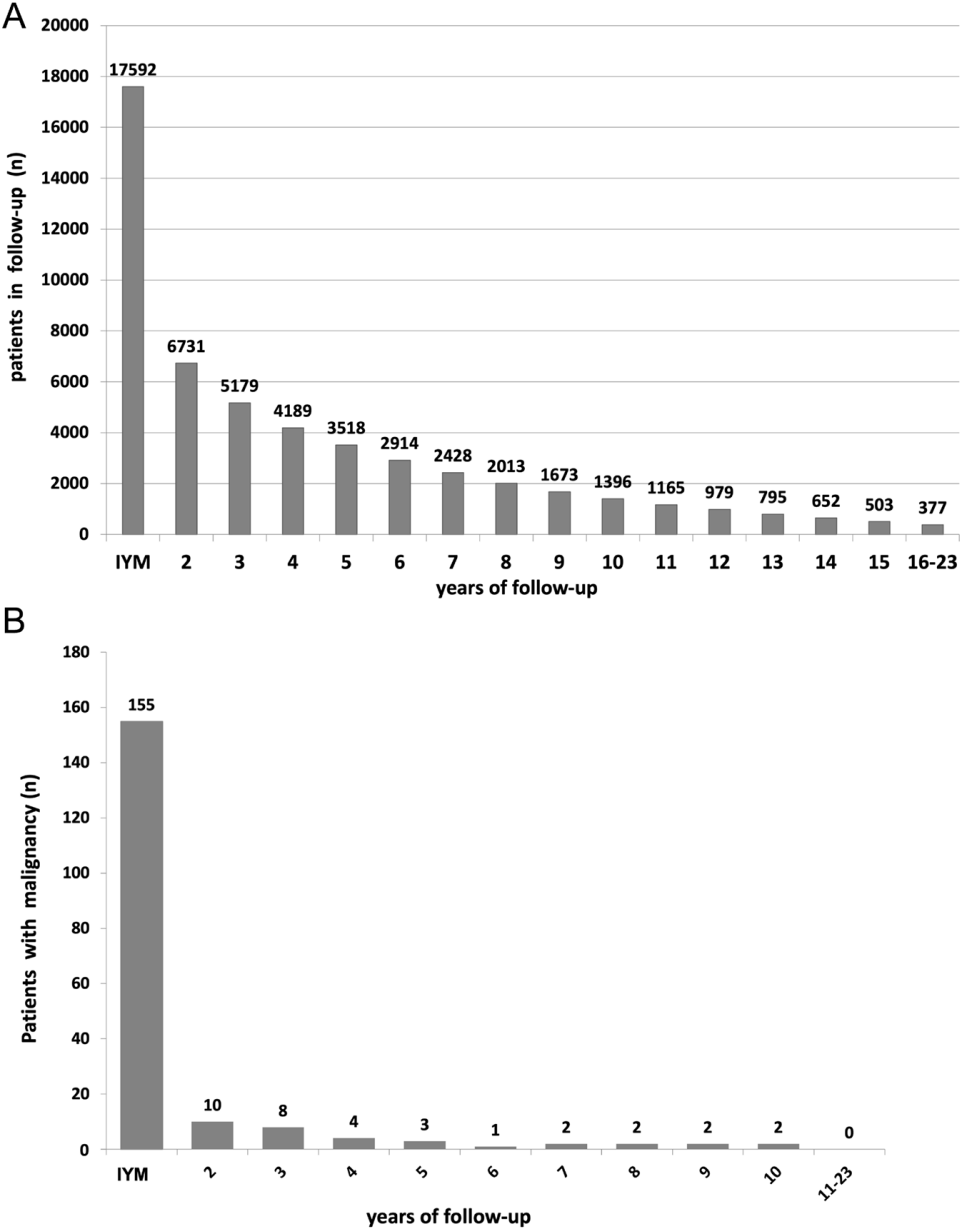
(A) Number of patients during initial year of management (IYM) and follow-up in years. (B) Number of patients with malignancies diagnosed during initial year of management and longitudinally followed/year.

Within the follow-up period (13–286 months after first visit, median 60 months), 34 malignancies (18 PTCs, 10 FTCs, 3 MTCs, 2 FVPTCs and 1 lymphoma) were diagnosed. A ROM of 0.14% was calculated during years 2–5 of follow-up, compared to 0.05% in years 6–10. Thereafter, no further malignancy was detected. Figure 2 details the size of the follow-up cohort/year (Fig. 2A) and the number of malignancies diagnosed per year (Fig. 2B).

## Discussion

Our data on patients with TN >1 cm represent one of the largest monocentric cohorts of patients with nodular thyroid disease managed by a standardized procedure and the largest one on longitudinal follow-up with more than 1000 patients followed for more than 10 years. The malignancy risk of only 1.1% is substantially lower than any previously reported (12, 13, 14, 15, 16, 17, 18, 19, 20, 21, 22, 23, 24) despite a high rate of cytological clarification in almost half of all patients. Our data allow a more precise estimation of the risk of malignancy in TN >1 cm patients first confronted with the diagnosis of a thyroid nodule and provide a more detailed insight into nodules with a low suspicion of malignancy on US and/or a benign rated FNAC.

### What is the explanation for our low ROM and for the discrepancies in relation to previously published data?

A simple and seemingly obvious explanation for the discrepancy may be the under-diagnosis of malignancy in our series. This seems unlikely, as our results on the accuracy of FNAC, performed in almost half of our patients, compare favorably with other series (12, 15, 17, 18, 19, 20, 21) (see Supplementary Tables 2 and 4). The reliability of our diagnoses is further supported by the low ROM over long-term follow-up where malignancies missed during the initial workup could be expected to surface in patients monitored for a sufficiently long time.

The exclusion of mPTCs in our series represents another clear difference from many previous studies. As guideline recommendations suggest that nodules >1 cm can safely be diagnosed (7), we deliberately focused on those nodules and excluded accidentally detected mPTCs (which would, however, have added no more than 0.2% to our total ROM).

The most likely explanation between our findings and previously published data is a different reference cohort. According to guideline recommendations, the dignity of a thyroid nodule ought to be stratified by sonomorphological and FNAC criteria (7, 8). Almost all larger published series report on operated nodules as exemplified by a meta-analysis of 32 studies worldwide by Singh Ospina *et al.* (22). They reported 4166 verified malignancies in 15,641 surgically treated nodules, corresponding to a ROM of 26.6%. This rate decreased to 5.2% when all 80,079 nodules were considered (22) (see Table 5). In comparison, the ROM of our cohort when restricted to surgically verified cases only is 11.9% but drops to 1.1% when all patients diagnosed with TN >1 cm are included. Thus, preselection of patients may explain part of the much higher ROM previously reported.

This effect may be amplified by a referral bias to the centers specialized in the management of thyroid malignancies. Due to their specialization, they will attract a higher proportion of malignant cases than exists in the general population ((12, 13, 15, 17, 19, 20, 21, 22, 23); for review see Table 5). A much higher ROM is to be expected in such a setting as compared to our institution where most patients first presented or were sent as a first referral point following initial diagnosis. A recent prospective, histologically verified, multicentric study from specialist thyroid centers in Germany indirectly supported this assumption of a preselection bias (25). The authors reported a mean ROM of 14% based on consecutively investigated patients with thyroid nodules. However, the prevalence varied significantly between participating centers, ranging from 0.5 to 30%. Interestingly, the center with the highest ROM was a center specializing in endocrine surgery.

### Are our results plausible in the context of known German data on the epidemiology of thyroid nodules and malignancy rates?

In large, US-based, epidemiological studies, the prevalence of thyroid nodules in Germany ranges from 35 to 60% (6). Only about a third of these cases were found to be diagnosed as part of the routine work-up by general practitioners (GPs) in a recent direct comparison of epidemiological and GP data of the same region (6, 26). GP diagnosis would thus reveal at least 8,000,000 patients with a thyroid nodule in a population of 83,000,000 in Germany. When we assume a ROM of 7–15% as suggested by guideline recommendations (3, 5, 7), about 560,000–1,200,000 German patients could be expected to be diagnosed with thyroid cancer. This sharply contrasts to the most recent 25-year prevalence of thyroid malignancies in Germany which averaged at only 103,300 (27) closely approximating the ROM of 1.1% calculated from our observation period of more than 20 years. Similar discrepancies can be extrapolated from data of other countries including the USA when we compare the expected rates of thyroid nodules to the thyroid cancer prevalence from cancer registries (28, 29, 30).

### What conclusions can be drawn from our follow-up data?

Only two previous longitudinal studies included a follow-up period beyond 5 years when starting at the time of diagnosis (18, 21) (see Supplementary Table 2). Ng *et al.* (21) described 279 diagnosed malignancies during follow-up of 2207 patients with FNAC. They diagnosed 82.5% of all malignancies during the first year after initial FNAC and 17.5% thereafter closely matching our findings. Additionally, both data (18, 21) support the accuracy of the current preoperative diagnosis using sonomorphological and cytological criteria (see Supplementary Table 2). As the first year most frequently represents the initial management, especially for difficult to diagnose patients, we deliberately set this period aside (IYM) and termed the follow-up period thereafter. ROM rapidly and progressively decreased during the first years of follow-up and no further malignancies were detected beyond 10 years of follow-up despite the large number of patients included (see Figs 1 and 2). These results obtained in all our patients are mirrored when the analysis is restricted to those patients initially classified as BC2. Their overall ROM (0.5%, or 1.3% when based on those 2202 patients with surgery or a minimum follow-up of more than 3 years) is much lower than recently described by Ng *et al.* (21) (ROM: 3.4%) but compares well to others ranging between 0.5 and 1.0% (18, 31) (see Supplementary Table 2) including a prospective study of Durante *et al.* (32) in 992 cases followed for 5 years (ROM: 0.5%).

Germany as a previously iodine-deficient area still has a high rate of multinodular goiter (6). Our longitudinal data may offer useful guidance for follow-up, as not all nodules with low suspicion on US will be sufficiently characterized in the initial workup and malignancy may only surface during long-term observation. This assumption would fit with the known slow growth rate of many low-grade, thyroid malignancies and is in line with the expected incidence of *de novo*-formed tumors (33, 34, 35, 36). Published guideline statements suggest that slow-growing tumors pose no additional risk and that US- guided follow-up of such nodules is dispensable (7, 37), but data supporting these clear-cut statements (for example, on long-term observation of thyroid nodular disease) are sparse and restricted to cohorts seen in specialized thyroid centers (18, 21). Our data on longitudinal follow-up in primary/secondary care thus provide the first direct supporting evidence.

### Strengths and limitations of our study

One of the major strengths of the present analysis is its focus on an unselected cohort of patients. This contrasts with previously published data from centers prone to overestimate the ROM of thyroid nodules due to preselection. Further strengths are the uniform approach to diagnosis and management over a very long period of time, the continuity in the team of endocrinologists and cytologists involved, and a large number of patients subjected to FNAC. Furthermore, we assigned a high proportion of patients to long-term follow-up, thereby minimizing the risk of wrong conclusions being drawn in cases where hidden malignancies failed to be diagnosed during the initial work-up.

There are, however, several limitations to our series, the first being the retrospective nature of the analysis. Alongside this, there are a number of technical issues related to its long duration: the quality of the US equipment at the beginning of the study was naturally inferior to that used 23 years later when, in addition, standardized US classifications evolved (38, 39, 40, 41). Strict diagnostic criteria like TIRADS before surgery are important but could neither be applied nor evaluated in terms of impact, as a TIRADS was first proposed in May 2009 (38) and EUTIRADS was introduced much later (2017, 4 years after our observation period (39)). Despite that, we applied all major criteria for sonomorphological suspicion of malignancy throughout the entire observation period but longitudinal quality control beyond these criteria was impossible and thus not included. This applied as well for the diagnosis of potentially pathological lymph nodes not easily detectable with the early US devices. However, in contrast to today’s praxis based on high-end US diagnosis, we added FNAC in almost half of our patients to clarify the dignity of the nodule.

We discharged patients after IYM only when there was no suspicion of malignancy by all sonographic and FNAC criteria. This explains the high number of patients remaining on follow-up, which was common practice in Germany for a very long period of time. Unfortunately, we lost a small number of patients during follow-up, but in retrospect a quantitative subanalysis of this cohort was impossible. Despite the importance of such data, lost patients to follow-up have not been quantified or analyzed in any of the published studies. Nevertheless, the impact of this definitely small fraction of patients lost to follow-up will be small and would not substantially alter our results.

Finally, our analysis from a single center may be prone to bias when compared to a multicenter study. It is, however, unlikely that any bias would impact our results because we clarified more patients by FNAC (with a high success rate; see Supplementary Table 1) and included a long follow-up period for a large part of the cohort. Both of these factors would tend to raise rather than lower the ROM.

### Implications for the counseling of patients

The diagnosis of a thyroid nodule often causes great distress in patients, owing to a fear of malignancy as recently highlighted in several studies (9, 10). Despite the recommendations in guidelines to discharge patients with low suspicion of malignancy (7), these concerns of the patients on the dignity of their thyroid nodule(s) are shared by many of their attending physicians. As clear long-term follow-up data have been lacking so far, common clinical praxis leads to many unnecessary follow-up investigations, repeated FNAC and to a substantial rate of unnecessary surgery to the thyroid (42, 43). The present cohort from a primary/secondary center is the largest series so far with a (monocentric) follow-up of more than 5 years and provides a first quantitative long-term assessment of the malignancy risks of these unselected thyroid nodules following their initial diagnosis. The low overall ROM may help to substantially reduce anxieties of the patients; additionally, the rapid decrease in ROM seen here during the first few years of follow-up may as well guide colleagues in their diagnostic and therapeutic activity (especially avoidance of operations ‘for safety’s sake’) and reassure them to discharge patients with low US suspicion early from follow-up.

## Supplementary Material

Supplementary Material

## Declaration of interest

The authors declare that there is no conflict of interest that could be perceived as prejudicing the impartiality of the research reported.

## Funding

This work did not receive any specific grant from any funding agency in the public, commercial or not-for-profit sector.

## Author contribution statement

M G: Data collection; I R: FNA – Cytology; M G, I R, G B: Analysis and interpretation of the data; M G, G B: Conception, drafting and design of the manuscript.
