# Supplementary material for: Malignancy rates in thyroid nodules: a long-term cohort study of 17,592 patients
Source: Eur Thyroid J. 2022 May 30;11(4):e220027. doi: 10.1530/ETJ-22-0027 (PMC9254276; doi:10.1530/ETJ-22-0027)
Supplement: Supplementary Material [file supplementary_material.pdf]

# Supplement

## Fine-needle-aspiration-cytology (FNAC): results and correlation with histology

**Elements included in this supplement:**

**Supplementary Methods**

**Supplementary Results and discussion**

**Supplementary Table 1:** Initial FNAC results of all 7,776 patients and their relation to histological findings in the subgroup of 1,193 patients who underwent histological verification

**Supplementary Table 2:** Calculation of negative predictive value (NPV) and positive predictive value (PPV) of all patients with FNAC in comparison to other data

**Supplementary Table 3:** Comparison of our results with other publications: Distribution of FNAC results between the classes of the Bethesda system

**Supplementary Table 4:** Comparison of our results with other publications: Rate of malignancy (ROM) in relation to initial Bethesda classification

### Supplementary Methods

7,776 patients (44.2% of all) were subjected to fine-needle-aspiration-cytology (FNAC). The percentage of punctured patients did not change significantly during the observation period.

Analysis of all patients included clinical and biochemical investigation, ultrasound, FNAC results and a histological work-up in all operated cases.

55 patients with sonographically suspect thyroid nodules refused FNAC initially but agreed to surgery.

FNAC-smears were air-dried, stained according to May-Grünwald-Giemsa, and evaluated by two pathologists throughout the study. All FNAC results of patients obtained before the

introduction of the Bethesda system for reporting thyroid cytopathology (TBSRTC) (1) were retrospectively reassigned to Bethesda classes (BC) 1-6.

790 patients underwent histological verification during initial year of management (IYM). 3,092 patients were followed longitudinally with a median follow-up of 5 year, of whom 403 were operated during follow-up (see Fig.1 of main paper).

**Calculation of positive predictive value (PPV) and negative predictive value (NPV) of FNAC-results**

PPV and NPV of initial FNAC results were calculated for each Bethesda class (BC). PPV was calculated for all malignancies, using the definition: true positive/true positives plus false positive results, and NPV was defined by true negative/true negative plus false negative results. For calculation of NPV we combined 1,193 operated patients and the subgroup of 1,841 unoperated patients with follow-up over more than 3 years (median follow-up 7 years) to calculate true negative results.

## Supplementary results and discussion

**Distribution of FNAC–results and correlation of Bethesda classes with histology (see supplementary Table 1):**

Histologic verification was sought in 900 patients following a single FNAC and in another 293 after repeated procedures.

A malignancy was confirmed in 160 patients including 98 papillary carcinomas (PTC), 31 follicular carcinomas (FTC), 9 medullary carcinomas (MTC), 5 follicular variant of papillary carcinomas (FVPTC), 4 anaplastic carcinomas (ATC), 10 metastases of an extrathyroidal malignancy and 3 lymphomas (see supplementary Table 1).

Out of additional 23 patients with a mptc the carcinoma was localized within the index nodule in 10 and outside in 13 patients, none of all patients with mptc showed lymph node involvement.

Our results for PPV and NPV of each BC fit well with those recently published by Sauter et al. (2) and Ng et al. (3), who also published follow-up data of their patients (see supplementary Table 2), Like them, we classified all nodule(s) of a patient who developed no sign of malignancy after a follow-up of longer than 3 years as truly benign and included them in the calculation of NPV.

Our data are qualitatively comparable to other seminal studies previously published (2-11) (see supplementary Table 3) but show some distinct quantitative differences, including a relatively low percentage of patients classified as BC3. This Bethesda class has recently been the subject of controversial debate, particularly as it is only moderately reproducible (2,12). The fact that our results had to be reclassified according to the Bethesda system, which was introduced years after the start of our data collection (1), could be another factor underlying the divergence of our results from those of similar studies.

The percentage of BC5 and BC6 cases was also lower in our cohort than previously reported (see supplementary Table 3). This may be due to the origin of our cohort in primary/secondary care where one would expect a lower rate of malignancy than in findings from referral centers for thyroid cancer (see discussion of main paper). In addition, due to the much lower quality of the ultrasound equipment initially used, we performed FNAC in a large number of patients as a safety measure to clarify the dignity of a nodule. This increased the number of nodules with benign cytology and reduced the relative percentage of malignant findings.

Altogether, initial cytology revealed a benign result in 5,839 cases (75.1% of all initial FNAC`s classified as BC 2). Of these patients, 550 were sent for surgery without further FNAC during IYM, predominantly for diagnosis of a multinodular goiter but also where there was a high suspicion of malignancy (5 histologically diagnosed as PTC, and 2 as FTC).
2,121 patients, initially classified as BC2, were discharged after FNAC because these results matched unsuspicious findings in ultrasound.

Repeated FNACs were performed in another 1,118 BC2 - patients. Of these, 213 underwent surgery, leading to a histological diagnosis of 5 PTCs, 7 FTCs and 3 FVPTCs.
The remaining 2,050 patients (35 % of all patients initially classified as BC2) were longitudinally followed, a subgroup of 1,449 of them being monitored for 3.1 - 23 years (median 7 years). A total of 7 malignancies (5 PTCs, 1 FTCs and 1 MTC) were diagnosed during follow-up.
The ROM of all our BC2 patients (5,839 patients) calculated as 0.5% (see eTable 1) or 1.3% respectively when we restricted the analysis to those 2,202 patients treated by surgery or followed for more than 3 years (see supplementary Table 2)). This agrees well with the small number of larger, closely controlled studies conducted previously (2-4,7,9-11) (see supplementary Table 4).

Patients classified as BC1 pose a difficult diagnostic problem. They are composed of cases with non-diagnostic results but have, by definition, plain cysts where cyst fluids and virtually acellular specimens have been aspirated (1). In total, we diagnosed BC1 in 21.1 % of all FNACs but this percentage dropped to 8.2 % when we excluded the large number of pure thyroid cysts without solid components. In 1,004 patients (12.9%) pure cysts were diagnosed and aspirates sent for subsequent cytology. Surgery was performed in 120 patients (1 cystic PTC, 1 FTC, 1 MTC). Our results confirm previous findings of a low ROM of plain cysts and, as no malignancies were detected during long-term follow-up, they support the recent critical discussion on their inclusion to BC1 (13).
In 633 patients a nondiagnostic (non-cystic) BC1 result was found (8.1% of all patients with initial FNAC`s). 70 patients were sent for surgery (without an additional FNAC on account of suspicious ultrasound) confirming a malignancy in 9 cases (7 PTCs and 2 MTCs); 5 malignancies (3 PTCs, 1 FTC and 1 lymphoma) were diagnosed after repetition of FNAC.

Histological clarification is advisable in BC3+4 cases and mandatory in all BC5+6 cases. Histology confirmed a malignancy in 5 of 26 BC3 patients (19%) and in 29 of 185 BC4 patients (16%), which fits well to previously published ROMs (see supplementary Table 4). In

18 of 27 patients classified as BC5 (67 %) a malignancy was histologically verified (3 patients of this group were lost to follow-up) and a malignancy was histologically confirmed in all 62 patients preoperatively classified as BC6, including the 10 patients with metastases (5 from lung cancer, 4 from a renal carcinoma and 1 from breast cancer).

**References:**


1. Cibas ES & Ali SZ. The Bethesda System for Reporting Thyroid Cytopathology. Thyroid 2009; 19; 1159–1165.

2. Sauter JL, Lehrke H, Zhang X , Al Badri OT , Rodriguez-Gutierrez R, Delivanis DA, Singh Ospina N, Donegan D, HamidiO, IñiguezAriza N et al.. Assessment of The Bethesda System for Reporting Thyroid Cytopathology. J Clin Pathol 2019;152(4):502-511.

3. Ng DL, van Zante A, Griffin A, Hills NK & Ljung B-M. A Large Thyroid Fine Needle Aspiration Biopsy Cohort with Long-Term Population-Based Follow-Up. Thyroid 2021; 31 (7): 1086-1095

4. Bongiovanni M, Spitale A, Faquin WC, Mazzucchelli L & Baloch ZW. The Bethesda system for reporting thyroid cytopathology: a meta-analysis. Acta Cytol 2012;56:333–339.

5. Krauss EA, Mahon M, Fede JM & Zhang L. Application of the Bethesda classification for thyroid fine-needle aspiration: institutional experience and meta-analysis. Arch Pathol Lab Med 2016;140:1121–1131.

6. Kim M, Park HJ, Min HS, Kwon HJ, Jung CK, Chae SW, Yoo HJ, Choi YD, Lee MJ, Kwak JJ, et al. The Use of the Bethesda System for Reporting Thyroid Cytopathology in Korea: A Nationwide Multicenter Survey by the Korean Society of Endocrine Pathologists. J Pathol Transl Med 2017; 51 (4): 410-417.

7. Liu Z, Liu D, Ma B , Zhang X, Su P , Chen I & Zeng Q. History and Practice of Thyroid Fine-Needle Aspiration in China, Based on Retrospective Study of the Practice in Shandong University Qilu Hospital. J Pathol Transl Med 2017;51(6):528-532.

8. Eszlinger M, Ullmann M , Ruschenburg I, Böhme K , Görke F, Franzius C, Adam S, Molwitz T, Landvogt C , Amro B et al. Low Malignancy Rates in Fine-Needle Aspiration Cytologies in a Primary Care Setting in Germany. Thyroid 2017; 27 (11):1385-1392

9. Reuters KB, Mamone MC, Ikejiri ES, Camacho CP, Nakabashi CCD, Janovsky CCP, Yang JH, Andreoni DM, Padovani R, Maciel RMB et al.. Bethesda Classification and Cytohistological Correlation of Thyroid Nodules in a Brazilian Thyroid Disease Center. Eur Thyroid J 2018;7:133–138.

10. Thewjitcharoen Y, Butadej S, Nakasatien S, Chotwanvirat P, Porramatikul S, Krittiyawong S, Lekpittaya N & Himathongkam T. Incidence and malignancy rates classified by The Bethesda System for Reporting Thyroid Cytopathology (TBSRTC) – An 8-year tertiary center experience in Thailand. J Clin Transl Endocrinol. 2019; 16: 100175.

11. Angell TE, Maurer R, Wang Z, Kim MI, Alexander CA, Barletta JA, Benson CB, Cibas ES, Cho NL, Doherty GM, et al. A Cohort Analysis of Clinical and Ultrasound

Variables Predicting Cancer Risk in 20,001 Consecutive Thyroid Nodules. J Clin Endocrinol Metab. 2019;104(11):5665-5672

12. Singh RS & Wang HH: Eliminating the "Atypia of Undetermined Significance/Follicular Lesion of Undetermined Significance" Category From the Bethesda System for Reporting Thyroid Cytopathology. J Clin Pathol 2011; 136 (6), 896-902

13. Kanematsu R , Hirokawa M , Higuchi M, Suzuki A, Aga H, Tanaka A, Yamao N, Hayashi T, Kuma S, Miyauchi A. Risk of Malignancy and Clinical Outcomes of Cyst Fluid Only Nodules in the Thyroid Based on Ultrasound and Aspiration Cytology. Diagn Cytopathol. 2020;48(1):30-34.

**Supplementary Table 1:** Initial FNAC results of all 7,776 patients and their relation to histological findings in the subgroup of 1,193 patients who underwent histological verification

| **Bethesda class** | **1** | **2** | **3** | **4** | **5** | **6** | **all** |
|---|---|---|---|---|---|---|---|
| **pts. with FNAC (No.)** | 1,637* | 5,839 | 26 | 185 | 27 | 62 | **7,776** |
| % of all pts. with FNAC | 21.1% | 75.1% | 0.3% | 2.4% | 0.4% | 0.8% | 100% |
| | | | | | | | |
| **pts. with FNAC + surgery (No.)** | **190** | **763** | **12** | **142** | **24** | **62** | **1193** |
| benign | 170 | 721 | 6 | 109 | 4 | 0 | **1010** |
| mptc | 3 | 13 | 1 | 4 | 2 | 0 | **23** |
| **malignancies:** | | | | | | | |
| PTC | 11 | 15 | 2 | 12 | 12 | 46 | **98** |
| FVPTC | 0 | 3 | 0 | 2 | 0 | 0 | **5** |
| FTC | 2 | 10 | 2 | 14 | 2 | 1 | **31** |
| ATC | 0 | 0 | 0 | 0 | 1 | 3 | **4** |
| MTC | 3 | 1 | 0 | 0 | 0 | 5 | **9** |
| malignant lymphoma | 1 | 0 | 1 | 0 | 1 | 0 | **3** |
| metastases of other malignancies | 0 | 0 | 0 | 1 | 2 | 7 | **10** |
| **all malignancies** | **17** | **29** | **5** | **29** | **18** | **62** | **160** |
| ROM/ BC | 1.0% | 0.5% | 19% | 16% | 67% | 100% | |

*BC1 results consists of 1,004 patients with plain cysts where cystic fluid was analyzed and 633 patients with non-diagnostic results

**Abbreviations:**

**ATC:** anaplastic thyroid carcinoma; **BC**: Bethesda class; **FNAC**: fine-needle-aspiration-cytology; **FTC:** follicular thyroid carcinoma; **FVPTC:** follicular variant of papillary thyroid carcinoma; **mptc:** papillary microcarcinoma; **MTC:** medullary thyroid carcinoma; **pts.:** patients; **PTC:** papillary thyroid carcinoma; **ROM/BC:** rate of malignancy per Bethesda class

**Supplementary Table 2: PPV and NPV per Bethesda class: comparison of our FNAC-data with recent publications**

| BC | our data | | | Sauter et al. 2019 (2) | | | Ng et al. 2021 (3) | | |
|---|---|---|---|---|---|---|---|---|---|
| | pts. (n)[1] | PPV | NPV | nod. (n)[2] | PPV | NPV | pts. (n) [3] | PPV | NPV |
| 1 | 582 | 2.9% | 97.1% | 202 | 3.5% | 96.5% | 236 | 6.8% | 93.2% |
| 2 | 2,202 | 1.3% | 98.7% | 945 | 1.5% | 98.5% | 1,575 | 3.4% | 96.6% |
| 3 | 14 | 35.7% | 64.3% | 20 | 15.0% | 85.0% | 135 | 24.5% | 75.5% |
| 4 | 150 | 19.3% | 80.7% | 147 | 11.6% | 88.4% | 107 | 29.0% | 81.0% |
| 5 | 24 | 75.0% | 25.0% | 70 | 87.1% | 12.9% | 20 | 70.0% | 30.0% |
| 6 | 62 | 100.0% | 0,0% | 232 | 98.3% | 1.7% | 134 | 100,0% | 0.0% |

[1] all patients with histological verification or follow-up of 3.1 - 23 years (median 7 years)

[2] all nodules (evaluated with TBSRTC) with histological verification or follow-up of 3 - 6.3 years (median 4.4 years)

[3] all patients with histological verification or follow-up of 10.5 -18.4 years (median 13.9 years)

**Abbreviations:**

**BC**: Bethesda class; **FNAC**: fine-needle-aspiration-cytology; **nod.:** thyroid nodules; **NPV:** negative predictive value; **PPV:** positive predictive value; **pts. :** patients; **TBSRTC:** The Bethesda System for Reporting Cytopathology

**Supplementary Table 3: Comparison of our results with other publications:**

Distribution of FNAC results between the classes of the Bethesda system

| Bethesda class | 1 | 2 | 3 | 4 | 5 | 6 |
|---|---|---|---|---|---|---|
| **this study** | **21.1%** | **75.1%** | **0.30%** | **2.4%** | **0.40%** | **0.80%** |
| Bongiovanni et al. (2012) (4) | 12.9% | 59.3% | 9.6% | 10.1% | 2.7% | 5.4% |
| Krauss et al. (2016) (5) | 10, .4% | 76.9% | 3.4% | 1.9% | 4.1% | 3.3% |
| Kim et al.(2017) (6) | 0.9 – 21.2% | 41.7 – 83.9% | 3.8 – 19.8% | 0 – 2.1% | 2.0 – 16.6% | 0.8 – 17.1% |
| Liu et al.(2017) (7) | 3.6% | 44.7% | 6.9% | 0.2% | 14.1% | 30.6% |
| Eszlinger et al.(2017) (8) | 39.4% | 48.4% | 6.6% | 3.9% | 1.0% | 0.7% |
| Reuters et al. (2018) (9) | 11.0% | 59.9% | 7.1% | 8.6% | 5.1% | 8.3% |
| Sauter et al. 2019 (2) | 10.5% | 72.8% | 0.8% | 5.5% | 2.4% | 8.0% |
| Thewjitcharon et al.(2019) (10) | 21.1% | 66,7% | 4,7% | 2,4% | 1,8% | 3,3% |
| Angell et al. (2019) (11) | 6.4% | 70.6% | 6.7% | 6.2% | 5.0% | 5.1% |
| Ng et al. (2021) (3) | 10.7% | 71.4% | 6.1% | 4.9% | 0.91% | 6.1% |

**Supplementary Table 4: Comparison of our results with other publications:**

Rate of malignancy in relation to the initial Bethesda classification

| **Bethesda class** | **1** | **2** | **3** | **4** | **5** | **6** |
|---|---|---|---|---|---|---|
| **this study** | **1%** | **1%** | **19%** | **16%** | **67%** | **100%** |
| Cibas &Ali (2009) (calculated) (1) | 1 - 4% | 0 - 3% | 5 - 15% | 15 - 30% | 60 - 75% | 97 - 99% |
| Bongiovanni et al. (2012) (4) | 17% | 4% | 16% | 26% | 75% | 99% |
| Liu et al.(2017) (7) | 0% | 34% | 71% | 67% | 69% | 97% |
| Reuters et al. (2018) (9) | 26% | 6% | 12% | 21% | 73% | 97% |
| Sauter et al. (2019) (2) | 4% | 2% | 15% | 12% | 87% | 98% |
| Thewjitcharon et al.(2019) (10) | 20% | 4% | 9% | 24% | 57% | 90% |
| Angell et al. (2019) (11) | 4% | 1% | 21% | 27% | 66% | 91% |
| Ng et al. (2021) (3) | 7% | 3% | 24% | 29% | 70% | 100% |
